# Supplementary material for: A Novel Synthesis of Poly(Ester-Alt-Selenide)s by Ring-Opening Copolymerization of γ-Selenobutyrolactone and Epoxy Monomer
Source: Polymers (Basel). 2020 May 25;12(5):1203. doi: 10.3390/polym12051203 (PMC7285084; doi:10.3390/polym12051203)
Supplement: Supplementary file 1 [file polymers-12-01203-s001.pdf]

Supporting Information for

A Novel Synthesis of Poly(ester-*alt*-selenide)s by Ring-Opening Copolymerization of γ-Selenobutyrolactone and Epoxy Monomer

Yanan Wang[1,2], Xiaofang Lin[1,2], Zhengbiao Zhang[1,2], Jian Zhu[1,2,*], Xiangqiang Pan[1,2,*] and Xiulin Zhu[1,2]

[1] State and Local Joint Engineering Laboratory for Novel Functional Polymeric Materials, College of Chemistry , Chemical Engineering and Materials Science, Soochow University , Suzhou 215123, China; Wangyanan20174@163.com (Y.W.); 20184209201@suda.edu.cn (X.L.); xlzhu@suda.edu.cn (X.Z.)

[2] Jiangsu Key Laboratory of Advanced Functional Polymer Design and Application, College of Chemistry, Chemical Engineering and Materials Science, Soochow University, Suzhou 215123, China

* Correspondence: panxq@suda.edu.cn (X.P.); chemzhujian@suda.edu.cn (J.Z.).

Table S1. Effect of different conditions on the Copolymerization of GPE with SBL[a]

Entry	Temperature (℃)	TBAB	Time (h)	Yield.[b](%)	M_n[c]/g mol^{-1}	*Đ*
1	0	5%	24	0	-	-
2	20	5%	24	36.2	2500	1.13
3	40	5%	24	61.5	5700	1.26
4	60	5%	24	85.7	6700	1.29
5	80	5%	24	98.6	8100	1.44
6	100	5%	24	98.6	6300	1.37
7	80	0.1%	24	90.5	8100	1.40
8	80	1%	24	98.5	8800	1.44
9	80	2%	24	98.5	8300	1.43
10	80	10%	24	98.1	6000	1.32
11	80	20%	24	97.0	5300	1.26
12	80	50%	24	96.1	3900	1.19
13	80	2%	0.5	24.5	2800	1.11
14	80	2%	1	38.1	3300	1.26
15	80	2%	2	74.5	5300	1.36
16	80	2%	6	97.6	7500	1.42
17	80	2%	12	98.1	8200	1.46

[a] The reaction was carried out with GPE (1.0 mmol) and TBL (1.0 mmol) using the TBAB in bulk. [b] Determined by ^{1}H NMR spectrum. [c] Estimated by SEC based on polystyrene standards.

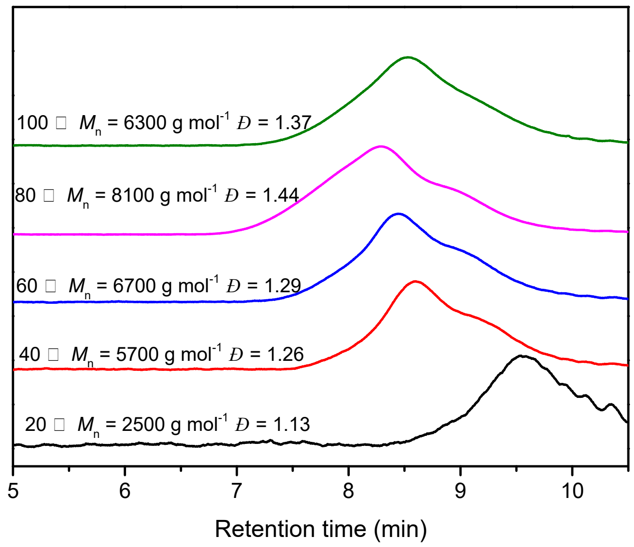

Fig. S1 Evolution of the SEC traces of the selenium-containing polymers with different temperature.

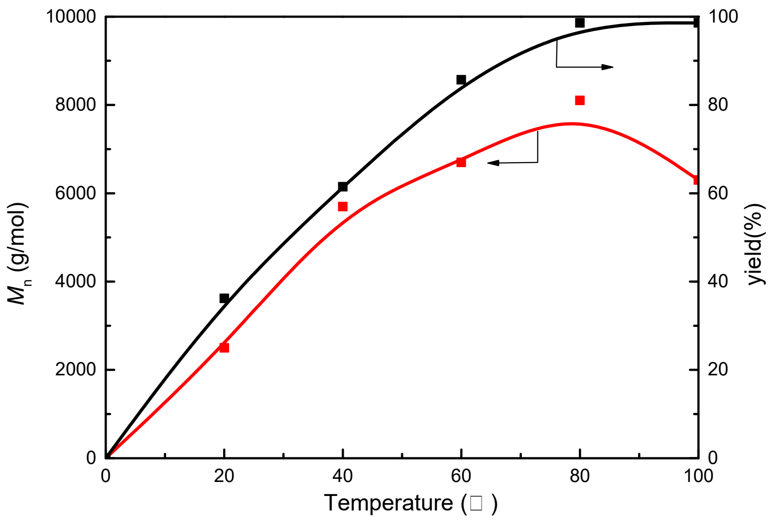

Fig. S2 Effect of reaction temperature on the copolymerization of GPE (1.0 mmol) with SBL (1.0 mmol) using TBAB (0.05 mmol) without solvent for 24 h.

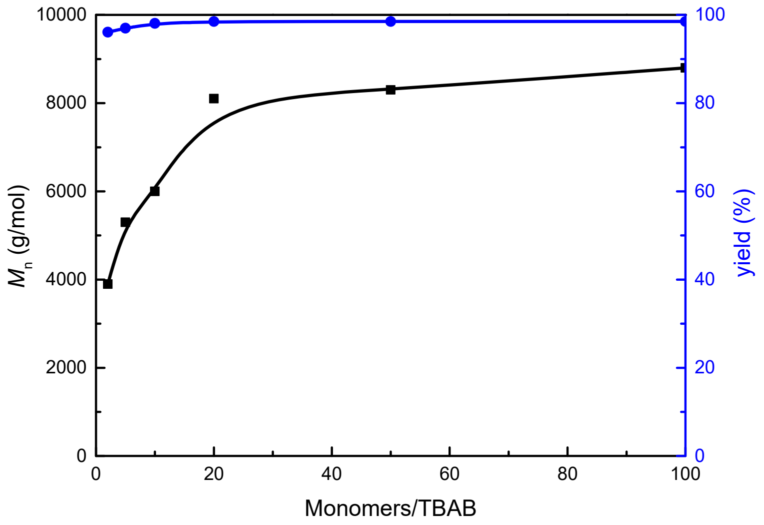

Fig. S3 Effect of feed ratio of monomers for TBAB on the copolymerization of GPE (1.0 mmol) with SBL (1.0 mmol) using TBAB without solvent at 80 °C for 24 h.

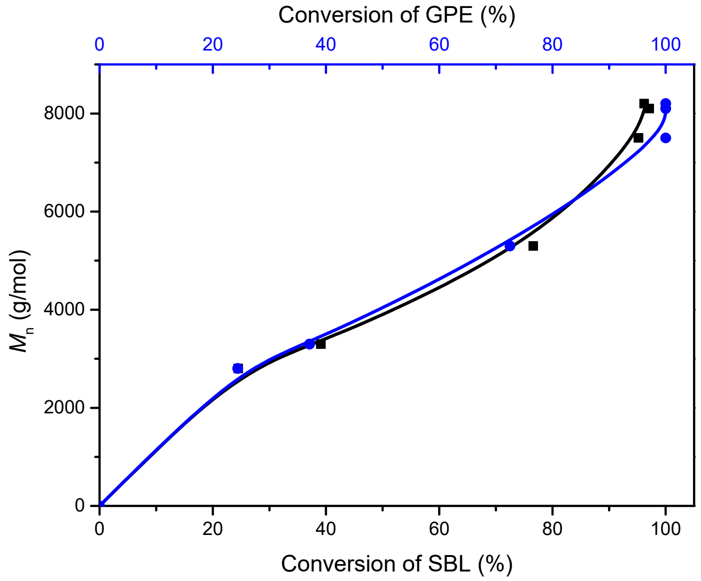

Fig. S4 Conversion-M_n of the copolymerization of GPE (1.0 mmol) and SBL (1.0 mmol) using TBAB (0.02 mmol) without solvent at 80 °C.

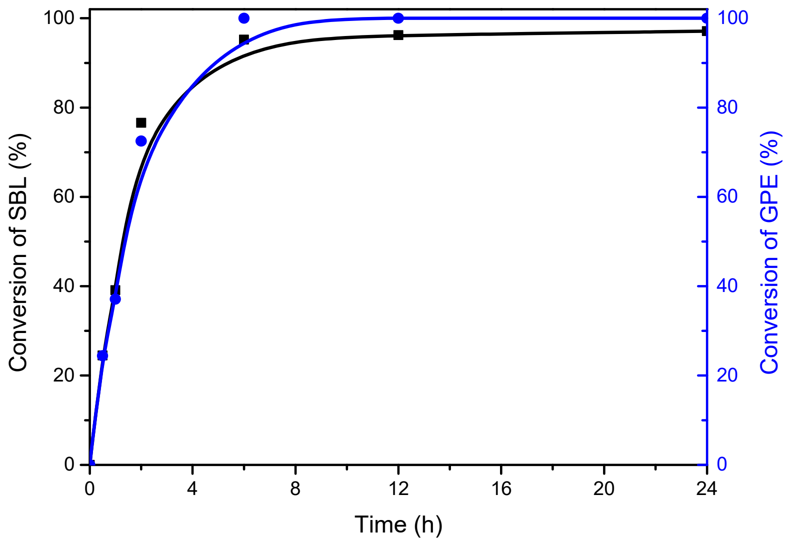

Fig. S5 Time-conversion of the copolymerization of GPE (1.0 mmol) and SBL (1.0 mmol) using TBAB (0.02 mmol) without solvent at 80 °C.

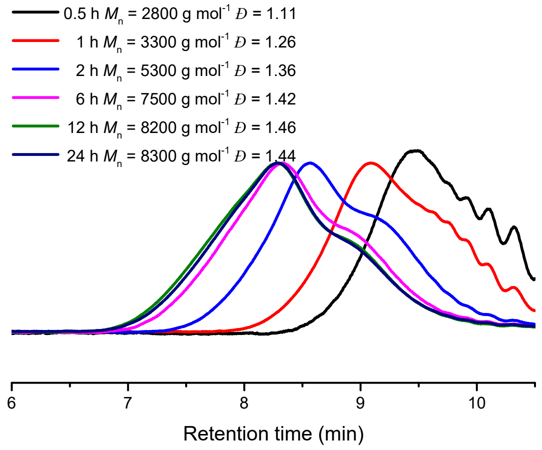

Fig. S6 Evolution of the SEC traces of the selenium-containing polymers with different time.

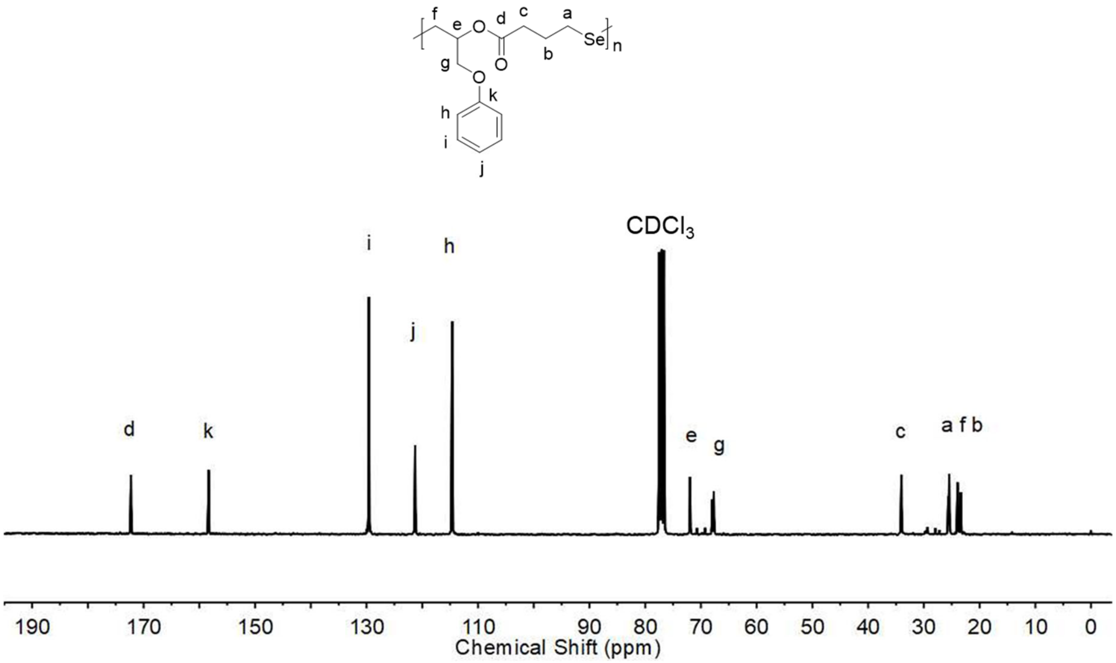

Fig. S7 ^{13}C NMR spectrum of PSe-1.

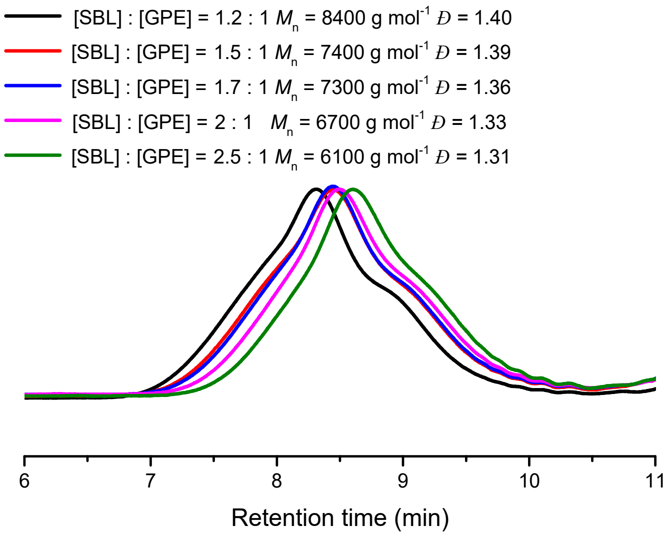

Fig. S8 Evolution of the SEC traces of the different ratio of the monomers.

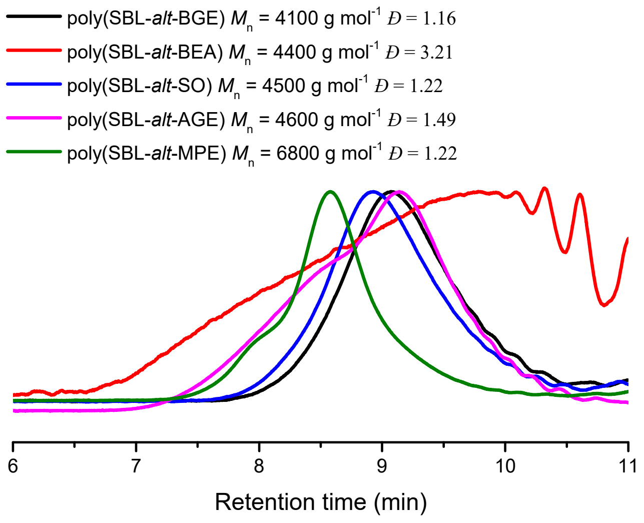

Fig. S9 Evolution of the SEC traces of the selenium-containing polymers.

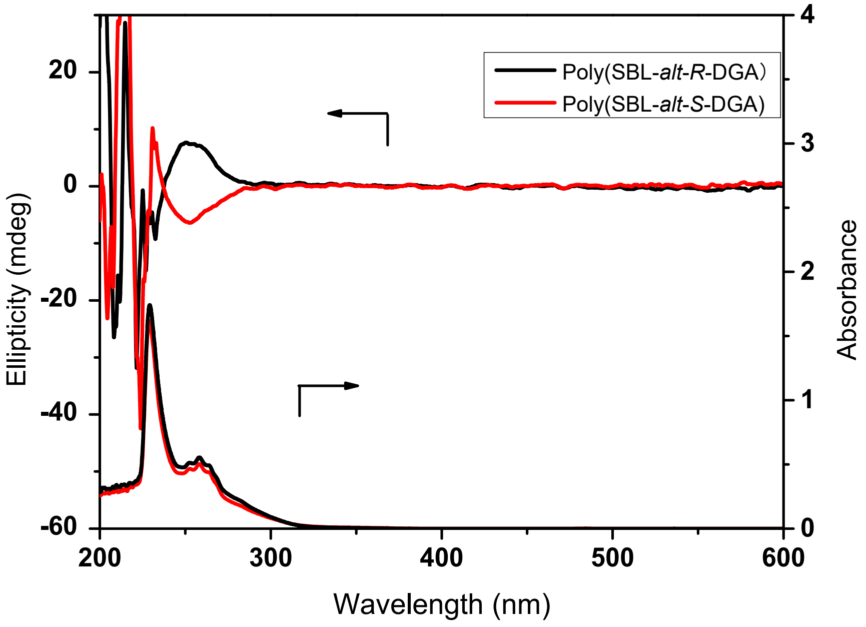

Fig S10. The maximum CD (260 nm) and UV-vis spectra in DCM.

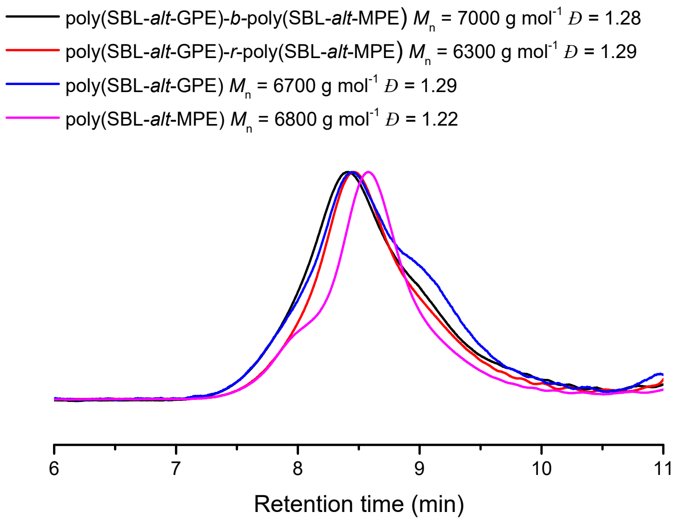

Fig. S11 Evolution of the SEC traces of the copolymer poly(SBL-*alt*-GPE)-*b*-poly(SBL-*alt*-MPE), poly(SBL-*alt*-GPE)-*r*-poly(SBL-*alt*-MPE), poly(SBL-*alt*-GPE) and poly(SBL-*alt*-MPE).

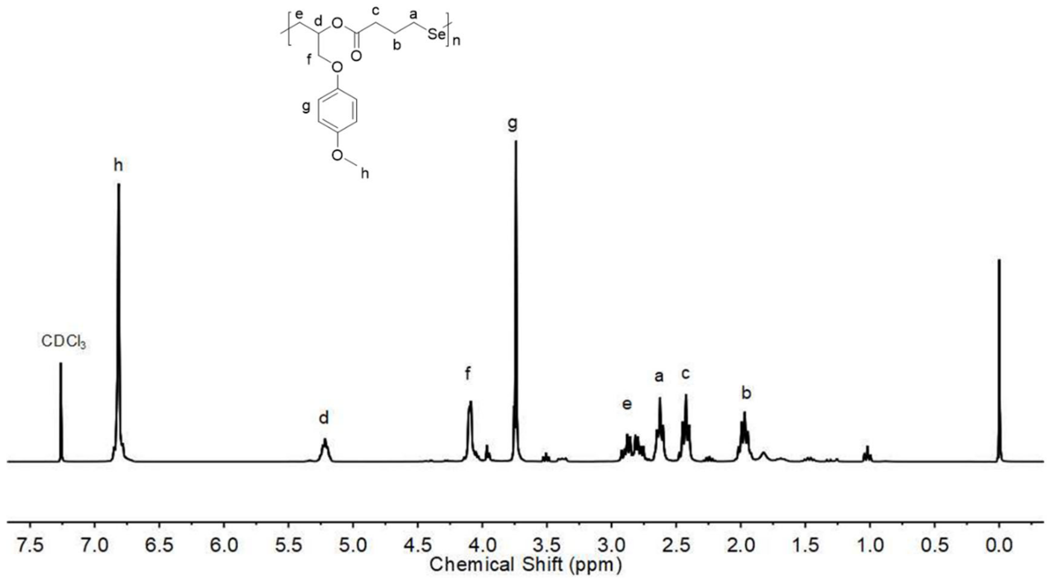

Fig. S12 ^{1}H NMR spectrum of poly(SBL-*alt*-MPE).

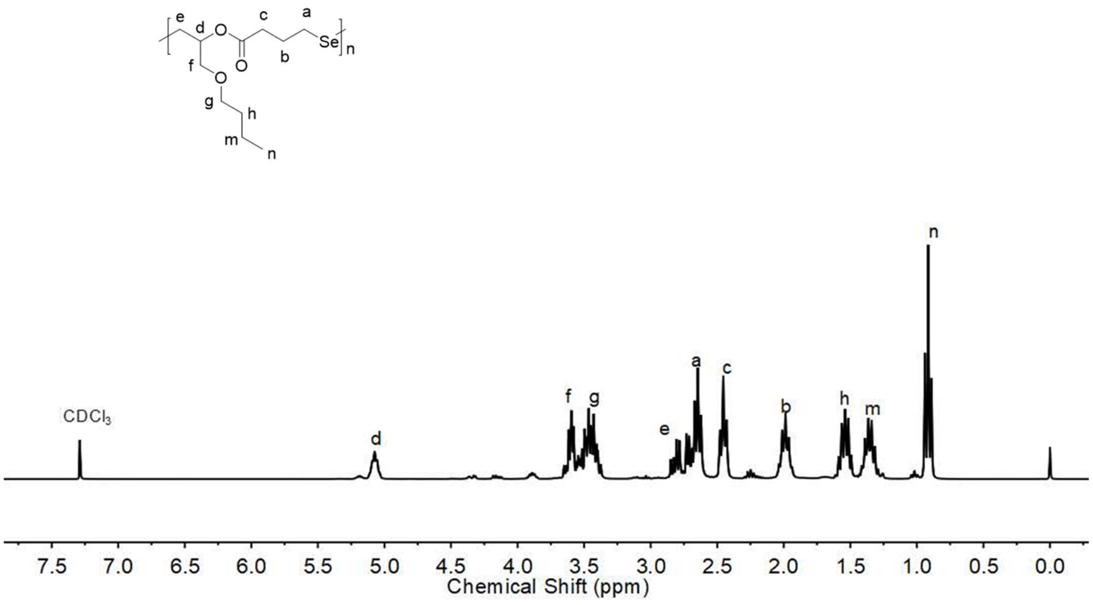

Fig. S13 ^{1}H NMR spectrum of poly(SBL-*alt*-BGE).

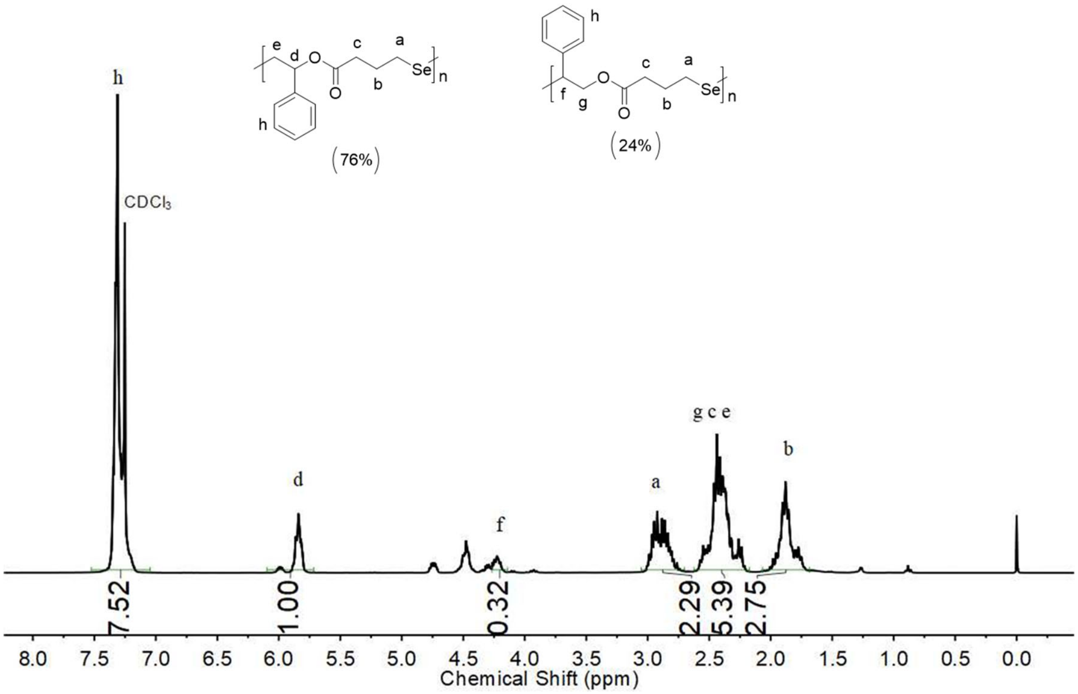

Fig. S14 ^{1}H NMR spectrum of poly(SBL-*alt*-SO).

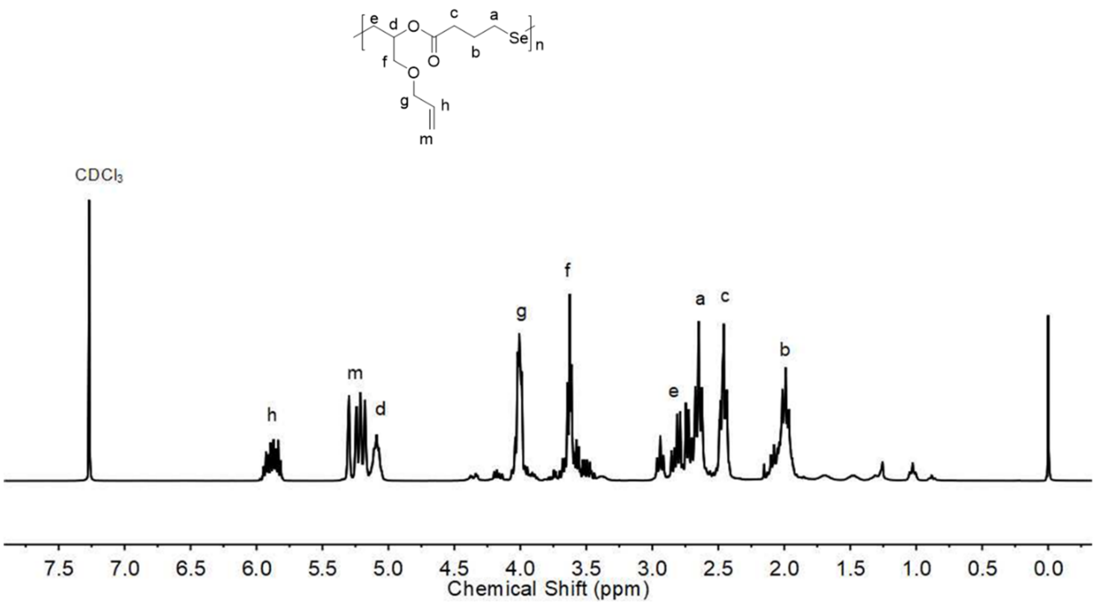

Fig. S15 ^{1}H NMR spectrum of poly**(**SBL-*alt*-AGE).

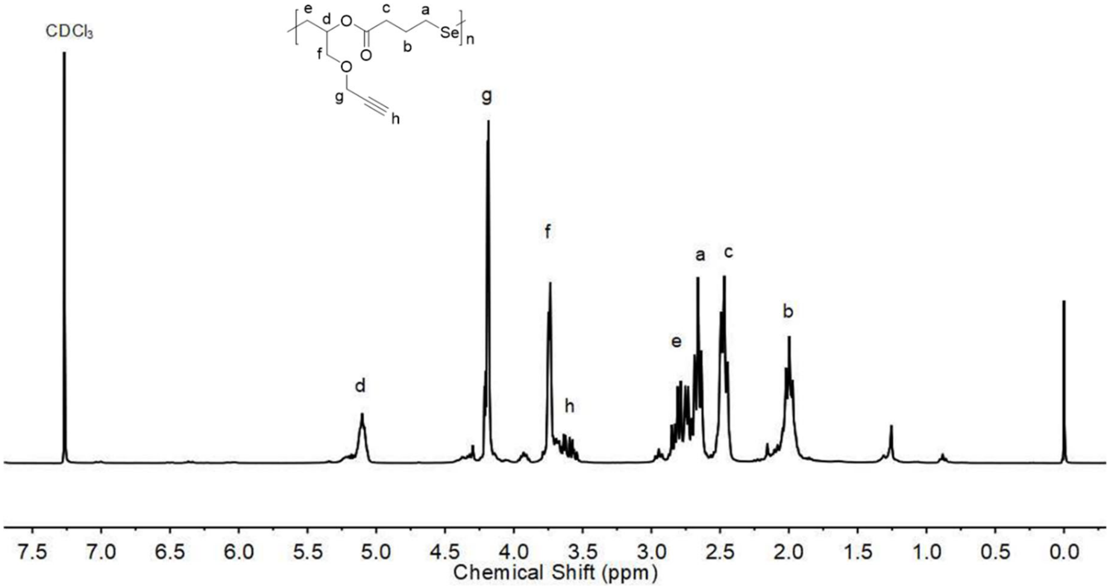

Fig. S16 ^{1}H NMR spectrum of poly**(**SBL-*alt*-BEA).

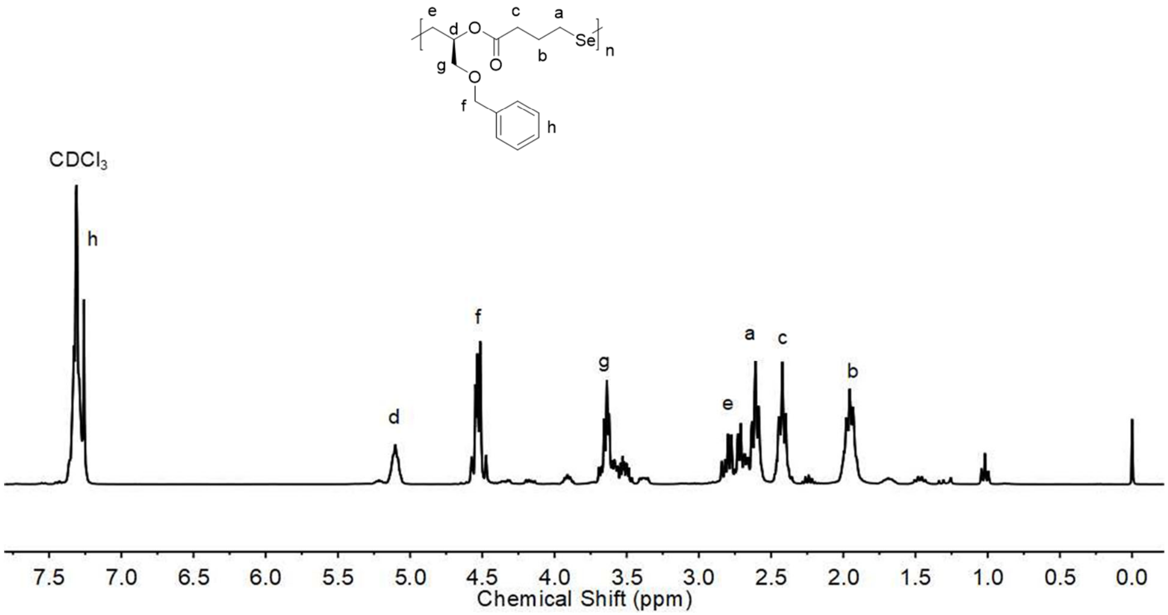

Fig. S17 ^{1}H NMR spectrum of poly(SBL-*alt*-*S*-DGA).

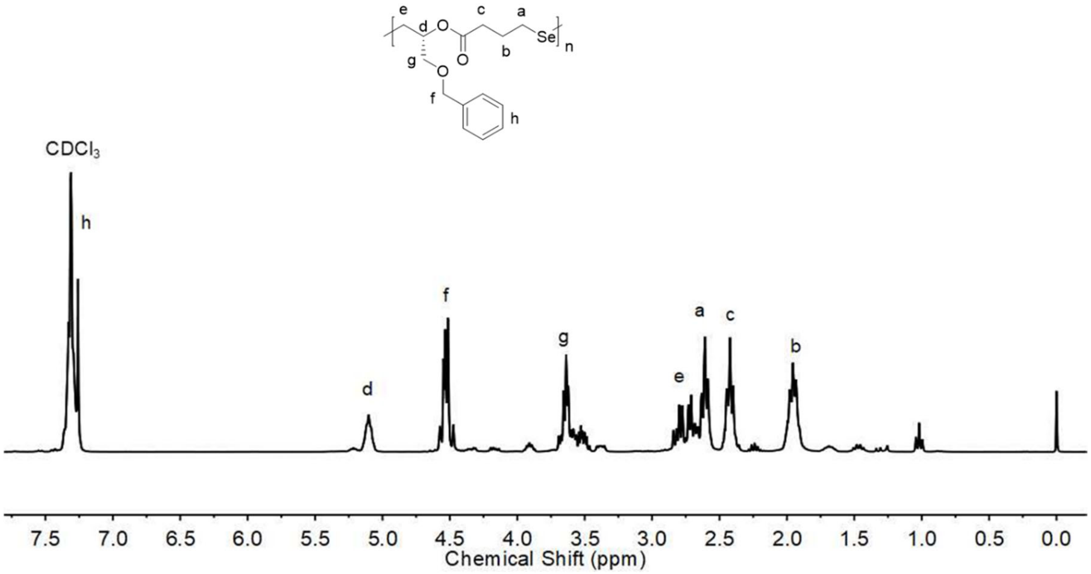

Fig. S18 ^{1}H NMR spectrum of poly(SBL-*alt*-*R*-DGA).

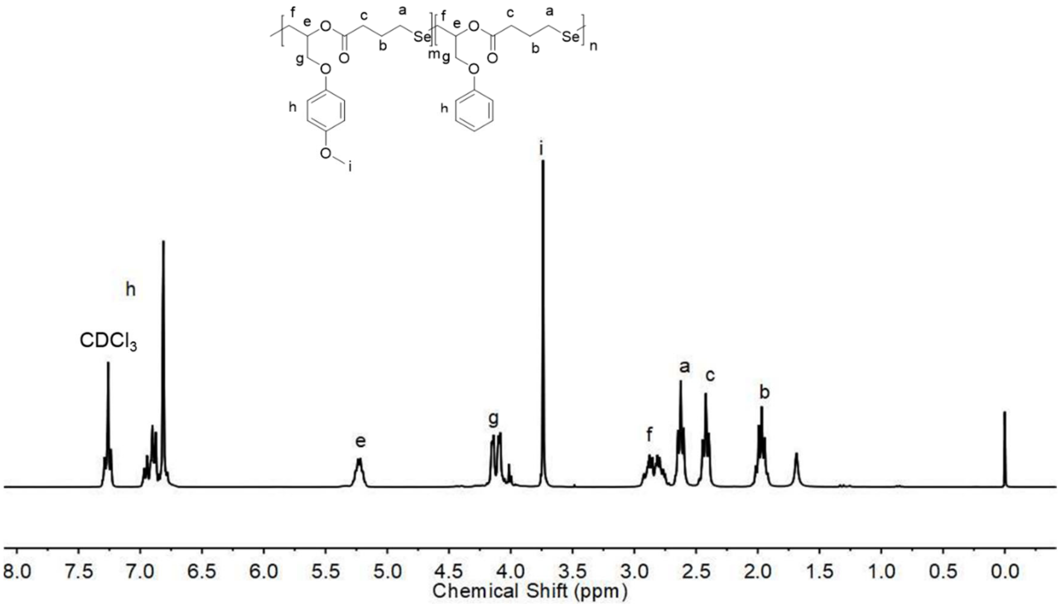

Fig. S19 ^{1}H NMR spectrum of poly**(**SBL-*alt*-GPE)-*b*-poly**(**SBL-*alt*-MPE).

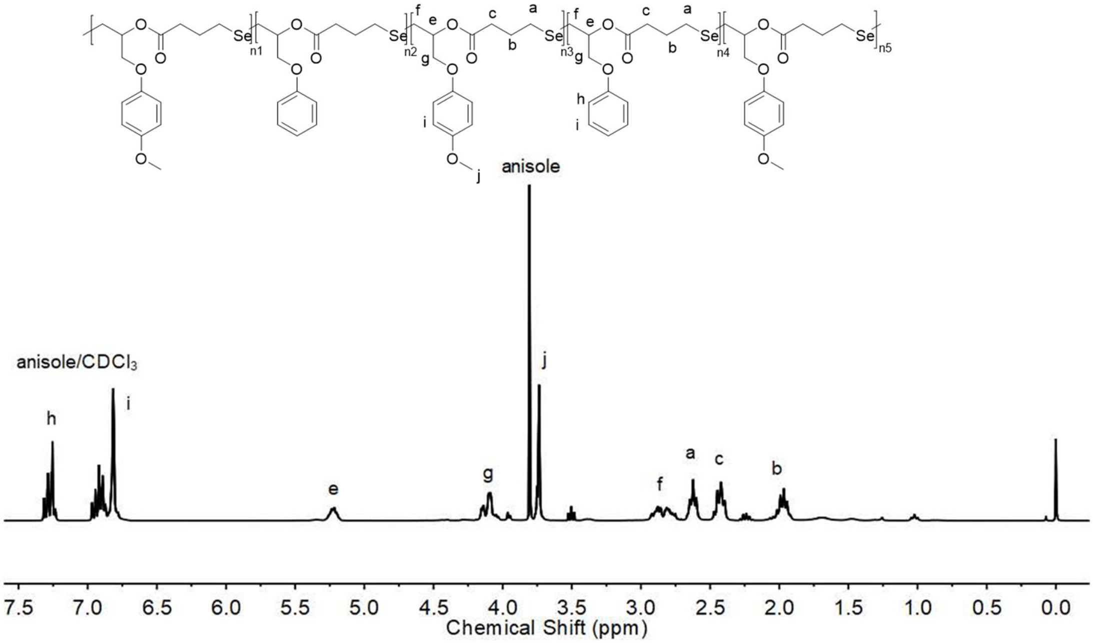

Fig. S20 ^{1}H NMR spectrum of multiblock copolymer.

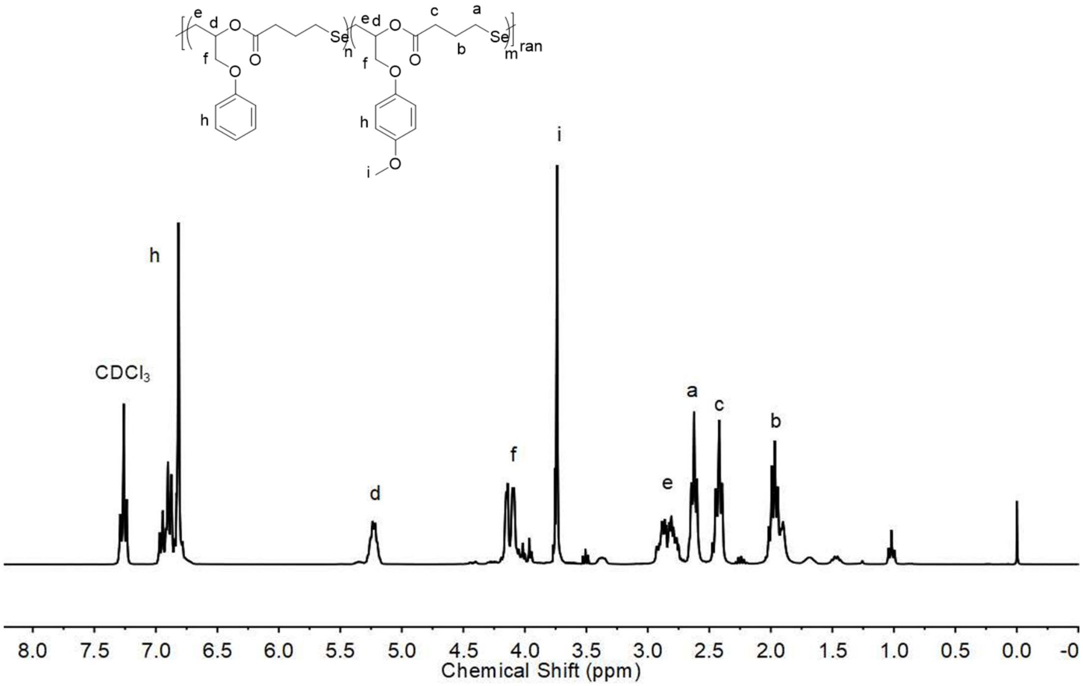

Fig. S21 ^{1}H NMR spectrum of poly(SBL-*alt*-GPE)-*r*-poly(SBL-*alt*-MPE).

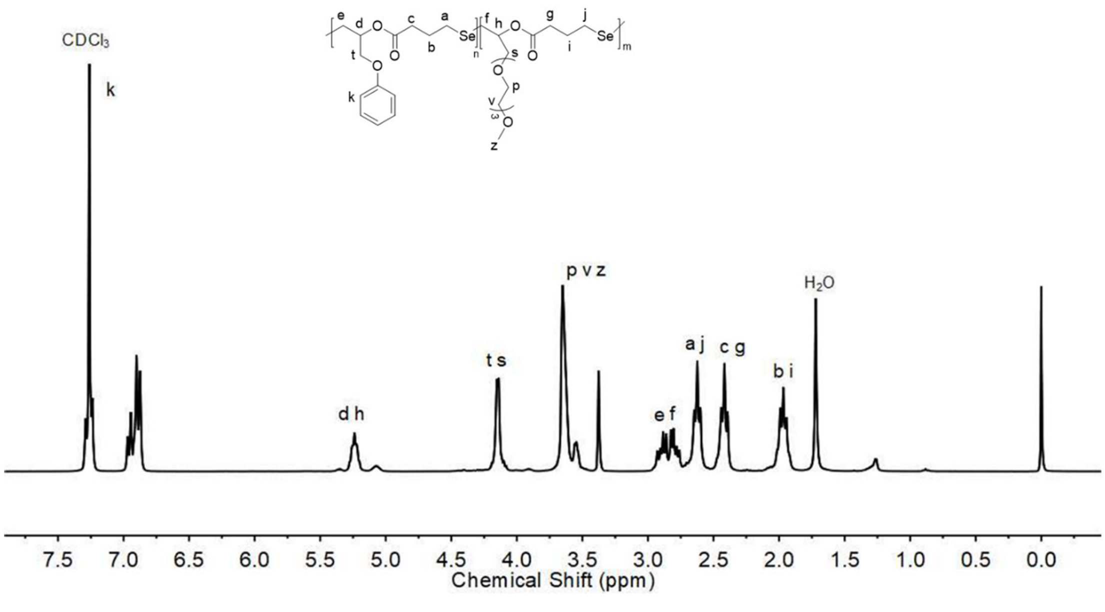

Fig. S22 ^{1}H NMR spectrum of poly(SBL-*alt*-GPE)-*b*-poly(SBL-*alt*-TGE).

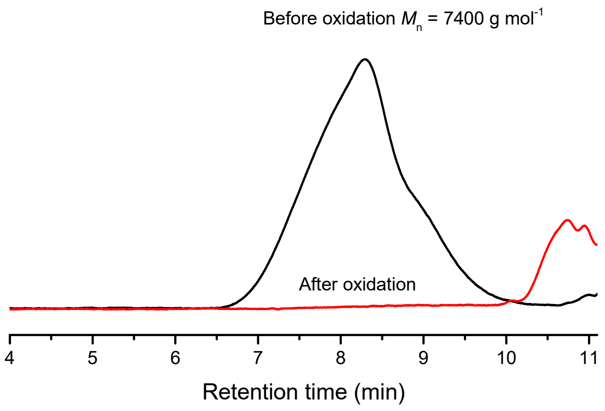

Fig. S23 SEC curves of poly(SBL-*alt*-GPE)-*b*-poly(SBL-*alt*-TGE) before and after treatment of hydrogen peroxide (0.1 M).
